# Preliminary Examination of an Appropriate Price Calculation Method and Medical Treatment Costs for Foreign Visitors in Japan

**DOI:** 10.3390/ijerph18115837

**Published:** 2021-05-29

**Authors:** Tomoyuki Takura, Study Group: Research on Appropriate Medical Treatment Prices for Foreigners Visiting Japan

**Affiliations:** Department of Healthcare Economics and Health Policy, Graduate School of Medicine, The University of Tokyo, Tokyo 113-8655, Japan; hehp-labo@umin.ac.jp

**Keywords:** market price, health insurance, foreign patients, cost accounting, pharyngitis, external injury

## Abstract

This study proposes a method for calculating the appropriate medical treatment price level for foreign visitors (FVs) in Japan. Hospital management costs and foreign prices were analyzed from a market principles perspective to determine the medical treatment price. The study involved two stages: a preliminary survey and an extended survey, supplemented by an international survey. Relatively frequent diseases were selected, and the costs incurred by hospitals for the treatment of FVs were analyzed though data from three hospitals, covering 24 outpatients and 4 inpatients. Payments made by three insurance companies for overseas medical institution services for Japanese tourists with pharyngitis were analyzed. This study shows that the appropriate medical treatment prices for FVs, considering profits, were 1.22–4.26 times higher compared with prices under Japan’s public health insurance plans. Furthermore, these prices were 1.31–4.26 times higher for outpatients with pharyngitis and external injury and 1.22–3.66 times higher for inpatients with appendicitis and femoral fractures. The price of pharyngitis treatment in 12 countries was USD 20.32–158.75 per patient for Japanese tourists, whereas FVs paid 60.24 dollars (1.13 times higher than Japan’s public healthcare price) in Japan. This study shows it was appropriate to set the ideal price level for FVs higher than that for Japanese patients.

## 1. Introduction

The Japanese government has set tourism by foreign visitors (FVs) as an industrial policy. In 2018, 31.19 million FVs visited Japan [1]; this number has since increased by 262%. Compared to 2018, the number of hospitals accepting FVs increased by 7.6% in 2019, and the annual number of accepted FVs increased by an average of 40.3% per medical institution [2]. The number of FVs in Japan has decreased by more than 80% due to the COVID-19 pandemic over the last year; however, it is estimated that the inflow of FVs will increase when the pandemic recedes due to the spread of vaccination and the acquisition of herd immunity.

Japan is set to host the Olympic and Paralympic Games in 2021 and various international exposition events in the near future, which are expected to attract many international travelers. Japan’s medical security scheme is characterized by the Universal Medical Care Insurance System. For proper operation of the National Health Insurance (NHI), keeping pricing systems under control is necessary to serve the increasing number of FVs who require emergency medical care due to accidents [3].

Japanese public health insurance is largely based on a fee-for-service payment system. This system, which is the basis of the national fee schedule for each type of medical treatment (inpatient, outpatient, and dental), applies to all Japanese citizens regardless of their occupation/income or gender/age group in the NHI. The fee-for-service payment system lists all services, procedures (e.g., surgery), and products (e.g., drugs) for medical treatment. In the NHI, regardless of the operational style of the medical facility (public/private), the amount billed by the medical institution for providing medical treatment—that is, the official price of healthcare services—is decided by the government.

Therefore, each medical institution has limited opportunities to independently consider the medical fee level, except for special medical services. Against this background, many medical institutions have not cultivated a system for setting prices. Owing to non-subscription to the NHI, FVs are not entitled to use medical care services under the same conditions as Japanese citizens. In other words, FVs are not locally insured patients. However, given the background mentioned above, it is believed that not all medical institutions have cultivated a system of setting prices for services for FVs. Therefore, it is necessary to determine appropriate pricing for the medical treatment of FVs in Japan. 

In general, the medical treatment fee for FVs is expected to be higher than the public fee under NHI as the following additional burdens are implied:

(1) Employment of coordinators, medical interpreters, and nurses/assistants who speak foreign languages.
(2) Physical infrastructure development (e.g., multilingual websites, in-institution medical guidance, and development of remote interpretation systems).
(3) Knowledge-oriented measures (e.g., manuals, checklists, and related education).
(4) Decrease in treatment efficiency (e.g., increased burden on medical care explanations, dispensing prescriptions, and risk countermeasures).

Burdens 1–3 are additional expenses that are not incurred by locally insured patients, only by FVs. These additional costs for FVs occur outside of fee-for-services payment in the NHI and, thus, it is necessary to set an appropriate price for them. For FVs, who are non-locally insured patients, the time requirement and resource consumption will increase due to various reasons, such as collecting medical information and carrying out test diagnoses, considering the risk of illness specific to their place of origin and race; formulating treatment plans and obtaining informed consent, considering FVs’ culture and religion; or conducting payment negotiations with an overseas insurer (burden 4: increasing basic medical expenses).

A survey by the Ministry of Health, Labour and Welfare reported that only 4% of the medical institutions (*n* = 4971) that responded had an FV billing price of JPY 20 (USD 0.18), more than double the fee for Japanese patients) or more per point in medical fee [4] (NHI: 1 point = JPY 10: USD 0.09). Most medical institutions are unable to consider the cost of additional treatment for an FV, which may affect their financial management and the stability of medical service supply.

Theoretical and economic evidence of the appropriate medical treatment price level for FVs in Japan is insufficient. The purpose of this study is to examine the medical treatment price to secure stable hospital management—a necessity for the stable provision of medical services to FVs. A method for calculating the medical treatment price level for FVs is therefore proposed.

## 2. Methods

### 2.1. Theories and Methods Related to Price-Setting Research

For price optimization, it is desirable to consider the behavior and motivation of market economic agents as well as the pricing mechanism for goods and services, including resource allocation and income distribution. Overall, the general economics approach is limited as there are various uncertainties related to the highly specialized technologies in medical science. Thus, examining price setting in the medical field is generally considered difficult due to the complex involvement of various factors.

The price-setting approach in medical treatment can be discussed in terms of two major categories: “market-based” and “input-based” [5]. The “market-based” approach determines the price level by considering the actual market price of medical treatment, while the “input-based” approach is based on the consumption of goods and services. Generally, in countries where the medical system is mature, it is presumed that prices are being formed in the public medical market using these approaches.

Meanwhile, there are discussions about approaches that explain the economic value of individual medical technologies (services). For example, from the standpoint of a medical provider (the supply approach), the methods of “technical difficulty” and “medical cost” are often selected from the viewpoint of quality evaluation and business management. Furthermore, from the standpoint of the payer (beneficiary), the methods of “patient outcome”, “economic performance”, and “willingness to pay” are often selected from the viewpoint of market and value evaluation [6,7,8,9].

Additionally, there are cases where certain preconditions are set for the use of these indicators. For example, in Japan’s universal health insurance system, most of the prices charged to public insurers by medical institutions are centered on the direct medical cost, which is based on the consumption of medical resources considering their clinical usefulness and hospital operability. Incidentally, technical fees (such as those from surgery), which are influenced by the doctors’ specialty, are considered as factors of technical difficulty. Furthermore, overseas (developed countries) market prices are referred to when determining public prices for pharmaceutical resources and medical devices.

**Figure 1:**
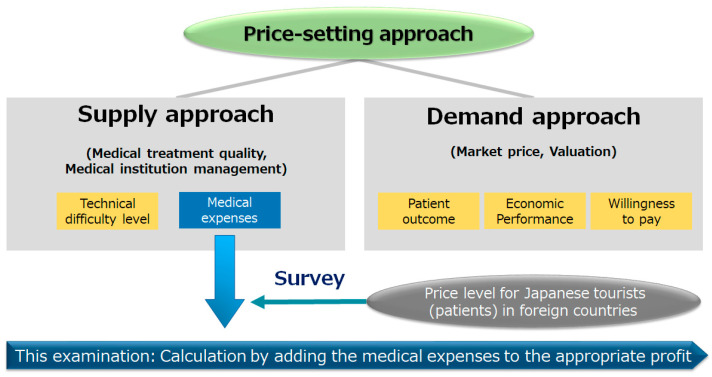
Theory of the price-setting approach (in general and within the range of this examination).

In this study, a method of setting the price level based on the analysis of medical expenses of Japanese medical institutions for FVs was examined, and international comparisons of price levels for Japanese tourists (patients) in foreign countries were conducted (Figure 1).

### 2.2. Price-Setting Research Centered on Medical Expenses

**Figure 2:**
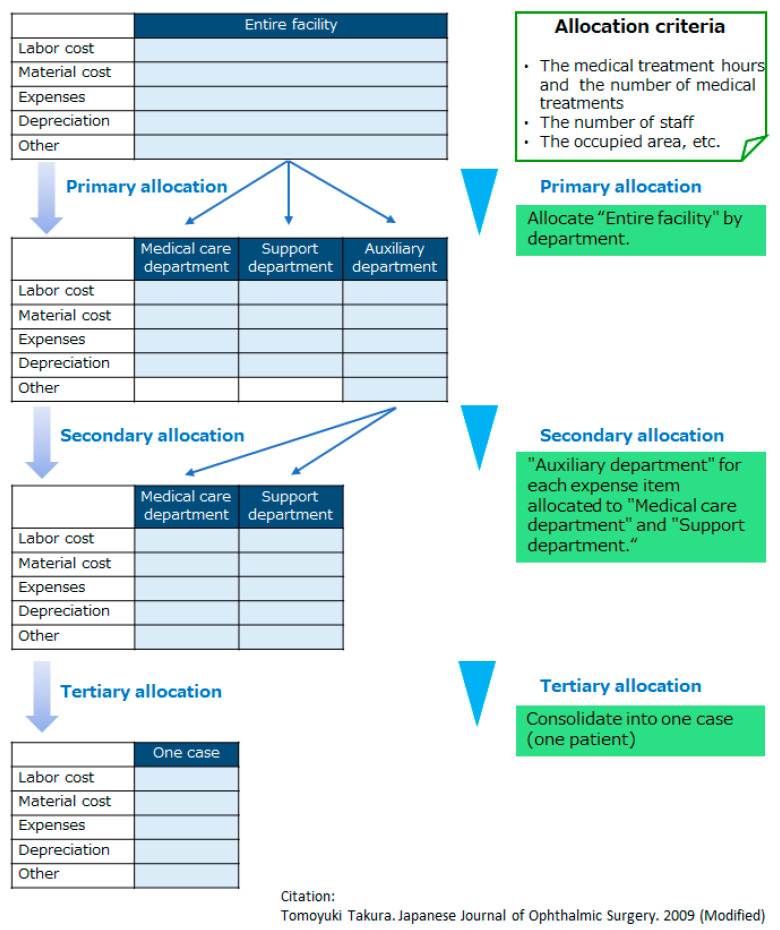
Cost of the entire facility apportioned to one case in three stages (calculation frame of medical expenses).

Cost is classified into actual and standard cost depending on the consumption and unit price calculation criteria. In addition, cost is divided into direct and indirect costs according to the consumption method and the association with medical practice. Both standard and indirect costs require an allocation/proration process to consolidate the consumption of medical resources into cost units based on a specific way of thinking. In this study, a method of setting prices based on the medical expenses per patient while referring to the cost accounting method of the Health Economics Research and Social Insurance Welfare Association, was adopted [10]. Specifically, the annual cost of the entire facility was apportioned to one case in three stages, using the number of medical treatments, the number of staff, and the occupied area as coefficients (Figure 2) [10]. The expense items were organized according to accounting items (resource consumption provided by medical treatments: labor costs, material costs, general expenses, depreciation costs, welfare costs). The billing items covered the medical treatments and related services.

In this calculation, the costs were analyzed based on socioeconomic ranges, taking the clinical characteristics and economic activities of an FV into consideration. Both the costs related to general medical care and the public investment in hospital management and healthcare infrastructure through the insurance system and various taxation systems that support Japan’s medical system, were taken into account. For example, the social insurance burden (insurance contributions and subsidies, such as operational grants to medical institutions) and additional expenses for FVs (e.g., interpretation costs, coordinator costs, equipment costs, and risk management costs) can be used as calculation items.

Three medical institutions with more than 400 beds were chosen as the target facilities, and their location conditions (urban or rural) were taken into consideration. In the calculation, additional factors (such as the occupancy and profit rate of each facility) were also considered. In this study, the data collection consisted of a medical practice survey and a medical institution management survey. The medical practice survey used the time study (occupation time of medical staff and institution equipment) and medical records (electronic and management ledgers). Some were self-reported alternatives based on professional experience. The medical institution management survey collected financial statements (profit and loss balance sheets), the number of patients and medical treatments, the number of staff and equipment, unit purchase price, and the area of each department.

**Figure 3:**
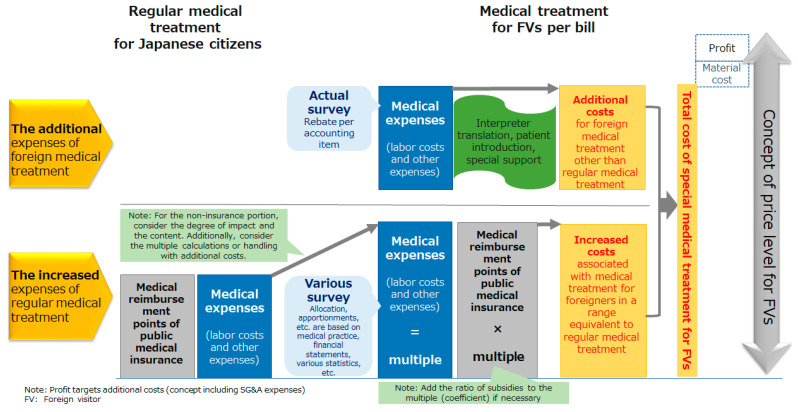
The structure of medical expenses for foreign visitors (FVs); calculation and addition from two perspectives.

The price of medical expenses for FVs was broadly divided into “additional expenses of foreign medical treatment” and “increased expenses of regular medical treatment” (Figure 3). The following definitions for additional and increased expenses were applied: The additional expenses were the costs for new, additional services (e.g., interpretation and transportation) for non-locally insured patients. The increased expenses were for medical services similarly offered to locally insured patients, but for non-locally insured patients, the unit price and quantity were increased (e.g., consultation hours and number of staff). Profit was included in this calculation as a necessary resource for reinvestment by medical institutions to realize sustainable management while appropriately responding to the medical needs of FVs. However, when determining the profit margins, the historical average of each institution was adopted to avoid distortion of price levels and the expensive economic burden on FVs due to excessive profits. In other words, the profits gained from FVs were basically the same as those gained from Japanese patients.

Regarding the “increased expenses of regular medical treatment”, the medical fee classification system (list) for public medical insurance was used to reduce the burden of cost accounting at each medical institution, improving the efficiency of billing operations and utilizing data across facilities (benchmark comparison standard). For the calculation unit of each medical practice, the unit price of medical treatment reward points (reimbursement amount) of public medical insurance was applied, the “increase coefficient” as the ratio to the medical expense for FVs calculated, and all prices were converted into multiples of the medical treatment reward points. The “additional expenses of foreign medical treatment” were also converted into a multiple of the medical treatment reward points. The “increase in the expense of ordinary medical treatment” and “additional expenses of foreign medical treatment” were added together to generate a bill. Finally, the medical material expenses, general and administrative expenses, and the institution’s average profits over two years were added. In addition, a part of the various unit price data (electric utility expense, water bill, wage unit price by occupation) was replaced by national designated statistics.

The above method was organized as a calculation manual (guidance), and a prototype calculation software was developed. The manual was published on the website of the Ministry of Health, Labour and Welfare in Japan [11]. The software was published on the homepage of the research group’s organization [12].

### 2.3. Stages of the Study

This study was conducted in two stages: a preliminary survey (2017–2018) and an extended survey (2019), supplemented by an international survey comparing medical treatment prices of representative disease. Preliminary research examined pricing methods and the feasibility of data collection. Based on these results, the number of cases was increased and an FV price analysis was validated in the extended survey. The extended survey focused on outpatient cases, considering the frequency of cases and the feasibility of conducting the survey.

As there were no available statistical surveys on the frequency of occurrence of FV diseases, this study was positioned as an exploratory report that also collected epidemiological information on the status of therapy for FVs. Furthermore, this study was conducted under the conditions of the availability of medical treatment data for FVs. Throughout the study, more than 10 target diseases (including pharyngitis, urticaria, cystitis, severe pneumonia, appendicitis, cholangitis, femoral fractures, asthma, external injury, and arrhythmia) frequently diagnosed for FVs in Japanese medical institutions were selected. These diseases were nearly identical to the representative diseases of Japanese medical treatment. Pharyngitis was the target disease in Section 2.4.

In the preliminary survey, seven target diseases (pharyngitis, urticaria, cystitis, severe pneumonia, appendicitis, cholangitis, and femoral fractures) which are frequently diagnosed for FVs in Japanese medical institutions were selected. In this study, supplemented by an international survey comparing medical treatment prices of representative disease, the consumption of medical resources was focused on dense equipment scale and allocation of human resources while paying attention to the balance between outpatient visits and inpatient treatments. However, statistical processing was difficult due to the sample size of this study.

The extended survey, covering 21 outpatients, used continuous observation (registration); the first-visit and second-visit samples were analyzed. The main target conditions were trauma and infectious diseases. Other cases included respiratory diseases, such as asthma and circulatory diseases, including arrhythmia. The inflow trends of FVs confirm that patients primarily come from North American, European, and Asian countries.

At both stages of the study, data from the same type of injury and medical intervention services were collected in parallel for Japanese and FV subjects. The public charges were calculated and compared to those for FVs. A Wilcoxon signed-rank test was employed to examine the mean population differences in this study. The statistical analysis software used was SPSS version 26.0 (IBM Corp., Armonk, NY, USA). The level of statistical significance was set at 5%, and mean values were expressed as standard deviations.

The calculation of costs may vary depending on how specific the diagnosis is, how much time a patient requires for recovery, and the occupancy rate of the medical institution. There were restrictions during sampling in this study. A one-dimensional sensitivity analysis was performed to verify the robustness and representativeness of the results for an extended survey, considering their uncertainty. In the sensitivity analysis, cases were first classified according to the International Classification of Diseases (ICD-10) and were then associated with clinical department.

The daily unit cost (clinic, outpatient, and Japan’s public healthcare unit price) and the national average value related to the number of patients by clinical department as per the government’s official statistics were then organized [13]. The weighting coefficient of the unit cost of medical treatment and the number of patients from the contents were determined, and the distribution of the difference between the medical cost for Japanese patients and that of the national average was calculated. Finally, for sensitivity analysis, the difference distribution of medical treatment costs of Japanese patients was compared with those of FVs when reduced by 25%, confirming the probability that the costs for FVs would exceed those for the Japanese.

### 2.4. International Comparison of Representative Medical Treatment Prices

It is necessary to discuss the following aspects related to the price level: (1) the estimation of profits for reinvestment, (2) patients’ payment ability for emergency services, and (3) subsidies for medical institution management. The risk of price negotiations and receivables has a major impact on management for medical institutions that are unable to refuse treatment due to regulations when the patient’s payment ability is low. Therefore, when examining the price level for FVs in Japan, it is desirable to refer to the price level and billing methods in foreign countries.

In this study, information on medical expenses in foreign countries was collected, focusing on high-prevalence diseases and medical departments that FVs most frequently consult. This survey investigated the level of payment for Japanese overseas travelers (overseas FVs) in overseas countries to evaluate the validity of the calculated medical treatment prices of FVs in Japan. The price level (representative cases), price breakdown (expenses, expense items, and ranges), and billing system (related payment systems) were also researched. The target areas were European countries whose healthcare systems, economic situations, and demographics are similar to those of Japan and other Asian countries, which account for a large percentage of FVs.

The main data were obtained from the medical expenses (self-funded medical treatment) for Japanese tourists during 2017–2018 from three insurance companies (private financial institutions and public payment agencies). Other statistical data were analyzed using public databases (PubMed data since 2010). Data collection was performed considering the payment system (self-pay, etc.), medical treatment structure (protocol, etc.), cost structure, and an analysis that aligned medical services and accounting expenses within a certain range. Data scrutiny was performed as much as possible, matching the periods to each other.

In order to ensure the accuracy of data comparison while considering various points to be noted for the target disease, pharyngitis (outpatient consultation) was selected based on the number of cases and type of medical treatment. Pharyngitis cases have a relatively high incidence, with little difference in medical care contents between countries (the initial diagnosis of pharyngitis, whether viral or bacterial, is relatively standardized).

The conversion of each country’s currency to American dollars was carried out using the exchange rate at the time of medical treatment. The average exchange rate between the Japanese yen and US dollar was 108.73 yen per dollar from 2017 to 2019. Since the proportion of services has increased in recent years, the significance of purchasing power parity (PPP) has decreased slightly. Regardless, there are many opportunities to use it, compared to the prevailing exchange rate. In this study, PPP-adjusted figures in US dollars are also shown [14].

## 3. Results

### 3.1. Appropriate Medical Expenses Based on Pricing in the Preliminary Survey

**Figure 4:**
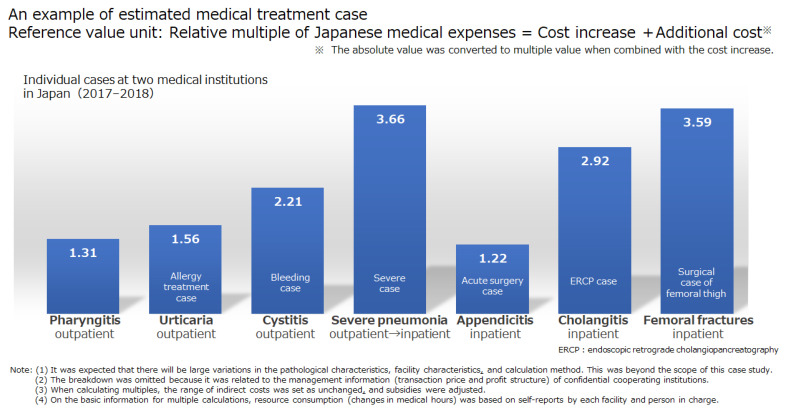
Calculation of price levels for foreign visitors (seven diseases).

As a result of the investigation, seven patients were selected: three outpatients with pharyngitis, urticaria (allergic treatment), and hemorrhagic cystitis and four inpatients with severe pneumonia (including outpatient treatment), general surgery for appendicitis, cholangitis treatment using endoscopy, and surgery for a fracture of the trochanteric region of the femur (including rehabilitation). This sample included one case that transitioned from outpatient to inpatient. The survey results are shown in Figure 4. The cost of medical treatment for Japanese (locally insured) patients was the average for the same cases in the past at the target hospital.

Compared with the medical expenses of Japanese patients, those for FVs were 1.31 times (1 point 0.12 dollars) higher for pharyngitis, 1.56 times (1 point 0.14 dollars) higher for urticaria with allergies, 2.21 times (1 point 0.20 dollars) higher for hemorrhagic cystitis, 3.66 times (1 point 0.34 dollars) higher for inpatients with severe pneumonia, 1.22 times (1 point 0.11 dollars) higher for general surgery of appendicitis, and 2.92 times (1 point 0.27 dollars) higher for endoscopic cholangitis treatment. The operating expense for the trochanteric fracture of the femur was 3.59 times (1 point 0.33 dollars) higher.

In summary, the medical expenses for FV patients were 1.22 to 3.66 times higher than those for Japanese patients, 1.31 to 2.21 times higher for outpatients (pharyngitis, urticaria, and cystitis), and 1.22 to 3.66 times higher for inpatients (severe pneumonia, appendicitis, cholangitis, and femoral fractures).

### 3.2. Survey for International Comparison of Medical Expenses (Self-Funded Medical Treatment)

**Figure 5:**
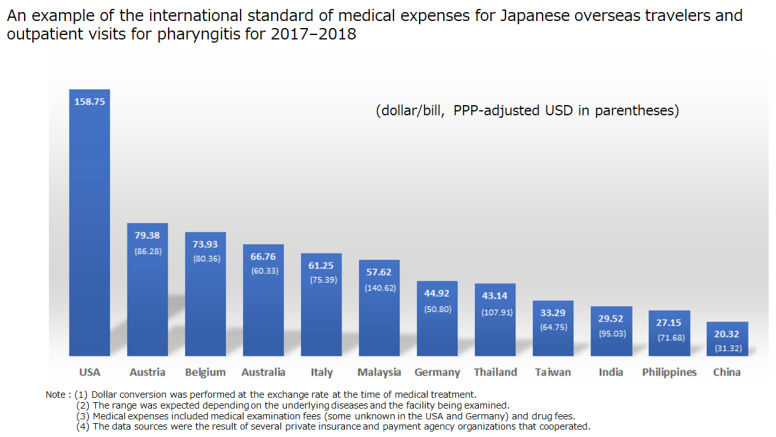
International comparison of medical expenses (pharyngitis and outpatient).

Figure 5 shows the amount billed when providing medical treatment to Japanese overseas travelers (overseas FVs) in each country. The survey indicates that although the total number of patients was 18 (one in each country other than USA, Australia, Italy, and China), the actual medical payment was about USD 20.32–158.75/bill (medical expenditures for medical examination and drug cost) in 12 countries (Figure 5). The highest price was in the United States, at USD 158.75/bill (medical fee may be partially unknown), followed by Austria with USD 79.38 (PPP: 86.28)/bill, and Belgium with USD 73.93 (PPP: 73.93)/bill. Western countries account for the top ranks of medical price levels. China has the lowest medical price level, at USD 20.32 (PPP: 31.32)/bill in, followed by the Philippines with USD 27.15 (PPP: 71.68)/bill and India with USD 29.52 (PPP: 95.03)/bill, since Asian countries generally have low medical price levels.

In this study, the results of the calculated actual cost for FVs were compared with the price levels for Japanese patients in foreign countries, and the validity of the price accounting method for FVs was confirmed. The general expenses for Japanese pharyngitis cases ranged from USD 41.00 to 46.00 for the initial consultation and medication in FY2018 (the initial consultation fee varies depending on the characteristics of the institution). However, the medical expense (excluding tax) for FVs, calculated by the price accounting method, was estimated 60.24 dollars in FY2018 (1.31 times higher than that for Japanese patients).

The results of this survey show that the actual expenses for the investigated FV cases were about the same as the actual billing amount (Japanese tourists) in the relevant country (Italy). In addition, the calculated price for the FVs was almost the average (58.00 dollars) for all surveyed countries.

### 3.3. Preliminary Evaluation of Pricing for FVs and the Difference between Calculated Price and Public Price in the Extended Survey

In the extended survey, the difference between the calculated FV price and the public price ranged between 1.95 (USD 32.83 vs. USD 64.18) and 4.26 times (USD 616.82 vs. USD 2626.31) in 23 cases (Table 1). This sample included 2 cases that were revisited repeatedly. This population had an average age of 51.4 ± 22.8 years and a male ratio of 33.3%. Comparing the prices of 23 FV and 23 Japanese cases, the cost for FVs was statistically significantly and 2.31 times higher than for the Japanese (USD 158.52 ± 156.52 vs. USD 445.61 ± 573.55, *p* = 0.029).

The smallest difference was for the diagnosis of suspected asthma (Ireland) and the largest difference was for the treatment of forehead contusion (Switzerland). Therefore, it can be inferred that the treatment price levels for FVs are higher by about 2 to 5 times the official price (outpatient cases only; ranged from 1.95 to 4.26 times).

As a result of the sensitivity analysis, the average ratio of medical treatment costs for FVs was 1.95 times higher than those for Japanese individuals, and the case rate of medical treatment costs for FVs exceeded those of Japanese by 60.0%. Therefore, it is speculated that the method of pricing for FVs adopted in this study is, to some extent, a valid approach. In addition, it is concluded that it is reasonable for the price level for FVs to be higher than the public price of Japanese patients.

## 4. Discussion

Medical care services for FVs in Japan have long been billed at the same level (public prices) as those of Japanese people since many medical institutions have few FVs and medical treatments. However, after 2015, the number of medical treatments increased sharply as the number of FVs increased [2]. As a result, the economic burden for FVs in hospital management has also increased; thus, it has become necessary to discuss appropriate FV pricing.

The medical treatment price level for FVs was identified as the sum of the “additional expenses of foreign medical treatment”, which includes the cost of items other than regular medical treatment and the “increased expenses on regular medical treatment”, which includes increases in the unit price of medical care for FVs within the range of regular medical care. It is believed that the method of calculating multiples of the current medical treatment reward points for Japanese individuals, based on this total expense, would be appropriate as a billing price for FVs. Profits were included in the calculation to allow for the sustainable management of medical institutions while appropriately responding to the medical needs of FVs in Japan. In other words, it was positioned as a necessary resource for the reinvestment of medical institutions and considered appropriate to incorporate the past average profit level for each facility into the calculation [15].

The contribution of the study findings would be valid only in a normal context, not considering the COVID-19 pandemic over the last year. It is therefore necessary to be careful in applying the results of this study to the management of hospitals under the current irregular circumstances. It is anticipated that the results of this study will be used in a meaningful way when the pandemic has subsided.

Future research should examine whether the calculation method in this study could be applied to other cases in which the medical treatment price level is specific because medical treatment expenses depend on the condition, age, and severity of the disease. Even if the disease is the same, the level of accounting cost is different; this applies not just to FVs but also to medical treatment for Japanese individuals [10]. Incidentally, the data in this study were collected under the conditions of availability. Therefore, there was a limitation on the representativeness of the findings. It is conceivable that the increase or decrease of more than a certain number of FV patients, that is, the productivity of medical services affects the medical treatment price. Unfortunately, due to data limitations, it is not possible to verify the impact of the result on increasing or decreasing FVs. This issue warrants long-term consideration.

It is important to examine whether the medical treatment expenses for FVs should be calculated individually based on the actual consumption of medical resources, treatment, and services. The advantage of this calculation method is that billing can be made according to the actual expenses. The disadvantage is that detailed data and calculations are required for each medical treatment case. As such, the expected price cannot be indicated to the patient in advance, which will hinder contract negotiations and cause accruals of outstanding amounts. Medical treatment expenses generally tend to be diverse depending on age, the severity of the disease, and the individual’s condition. It should be noted that this is not limited to FVs and can be considered the same in medical treatment for Japanese people. The regular price list is calculated based on typical treatment levels. The medical expenses of public medical insurance (1 point = JPY 10) can be regarded as the standard medical treatment price in Japan. This study effectively utilizes the public medical insurance claim system (unified prices for the whole of Japan), which comprehensively covers many medical treatments and improves the accuracy and efficiency of the pricing approach for FVs.

When considering the price setting of an FV, it is necessary to clarify the following: how many FVs need to receive medical treatment in Japan, what medical resources can be used, and how the medical treatment for FVs impacts the overall medical institution balance. Medical services, medical treatment explanations, price-contract negotiations, and planned price explanations may be requested in advance for FVs to avoid subsequent issues, such as receivables. In examining the medical price for FVs, it is essential to consider the operational expenses of medical facilities. The medical institution’s balance of income and expenditure is determined by the profit for each treatment case (difference between cost and price). If medical institutions have a higher number of FV patients, it can be assumed that their cost–income ratio is balanced by setting price points for standard medical treatments and applying the points of public medical insurance (converting one point to several tens of yen). By setting this standard price, which is the appropriate price based on medical expenses, medical institutions will increase their ability to explain price levels to FVs. Simultaneously, medical expenses can be presented before a doctor consultation so that problems in receivables are reduced, the balance of medical management is stabilized, and administrative procedures become more efficient.

When referring to the level of medical treatment prices overseas, not only should the real economy (economic level) of each country but also the medical system (comprising patient access, the composition of financial resources, and the degree of self-pay), history, and culture require special consideration [16,17]. The price in this study is a free price (self-pay medical treatment fee). Therefore, even for the same medical service, the payment amount generally differs depending on the solvency (economic power), cost level in the home country (affordability), and the amount billed by the medical institution.

It is necessary to consider the economic balance at the national level as well as the hospital level to recover public capital related to medical treatment for FVs in Japan. In this regard, it was speculated that the public expenditure portion of medical fees (used for general financial resources) would be appropriate for FVs in Japan by using the multiple calculation methods. Specifically, in this method, the portion corresponding to the public expenditure will also be paid by FVs who become patients (which is several times the amount that is paid by the Japanese). Regarding public infrastructure, taxes and indirect contributions to regional medical care should be considered. Examining the medical treatment price level for FVs also has the following benefits: (1) it ensures high returns to the national treasury through the consumption and business tax on self-funded medical treatment; (2) it contributes to regional medical care (residents) by strengthening the base of medical institutions that accept FVs; and (3) it boosts the stabilization of the employment of medical personnel related to FVs. Based on the results of this study, it is planned to develop a larger and more extensive survey and to develop an improved calculation manual for medical institutions.

This study was conducted based on the hypothesis that the provision of medical services to FVs consumes more medical resources than for the Japanese, considering the actual situation (increased burden) of medical practice in Japan. To test this hypothesis, it was also meaningful to confirm that the medical treatment price for foreigners overseas is higher than the price for the inhabitants of that country. Based on the results of this research, it is hoped that research on this subject will progress in the future.

## 5. Conclusions

This study identified various issues and directions regarding the setting of medical prices for FVs in Japan. Based on trial calculations of medical expenses and an overseas price survey, our study shows it is appropriate to set the ideal price level for FVs higher than that for Japanese patients. Furthermore, it is necessary to set standard prices for each medical institution and calculate the expected consumption of medical resources to control the balance of income and expenditure in hospital management while considering the total inflow of FVs into Japan. Based on this information, it is essential to provide prior explanations and follow-up measures to patients, families, and insurers.

## Figures and Tables

**Table 1:** Difference between calculated price of FVs and public-insurance price for Japanese nationals in the extended survey (including re-examination).

Country of Citizenship	Diagnosis	Medical Treatment Price (Dollar)		A multiple of the Japanese Medical Treatment Price
		Japanese Public Insurance Costs 100%	Foreign Visitors (FV) to Japan	
Ireland	Asthma (suspected)	32.83	64.18	1.95
USA	Urinary retention	151.47	420.73	2.78
USA	Atherosclerotic cerebral infarction/suspected acute phase	424.42	863.05	2.03
England	Pleurisy	66.68	133.82	2.01
England	Left knee joint pain	62.17	135.17	2.17
Israel	Arrhythmia	74.68	195.91	2.62
Israel	Head cut	84.06	196.39	2.34
Israel	Sciatica	61.89	150.67	2.43
Australia	Cystitis	51.96	131.07	2.52
Australia	Chief complaint: respiratory distress (no diagnosis)	47.64	93.92	1.97
Singapore	Lumbar compression fracture (suspected)	367.59	1309.58	3.56
Singapore	Swelling of the face	224.95	506.07	2.25
Switzerland	Forehead contusion	616.82	2626.31	4.26
Switzerland	Forehead contusion	213.73	667.96	3.13
Thailand	Insect bites/Phlebitis (suspected)	99.78	211.90	2.12
Taiwan	Suspected influenza	117.16	321.82	2.75
Taiwan	Left head bruise	249.41	654.78	2.63
Taiwan	Head flapping, chin cut	405.57	841.22	2.07
China	Croup syndrome	103.92	218.62	2.10
China	Pregnancy, irregular bleeding	74.68	149.54	2.00
Germany	Acute cystitis	43.04	159.36	3.70
Germany	Acute cystitis	38.81	129.08	3.33
France	Gout	3560	7392	2.08

## Data Availability

The data collected for this study are highly sensitive, and if reasonably requested, the data supporting the findings of this study can be obtained from the project director.
